# Canonical and non-canonical EcfG sigma factors control the general stress response in *Rhizobium etli*

**DOI:** 10.1002/mbo3.137

**Published:** 2013-10-28

**Authors:** Ann Jans, Maarten Vercruysse, Shanjun Gao, Kristof Engelen, Ivo Lambrichts, Maarten Fauvart, Jan Michiels

**Affiliations:** 1 Centre of Microbial and Plant Genetics, KU LeuvenHeverlee, B-3001, Belgium; 2 Biomedical Research Institute, Hasselt UniversityDiepenbeek, B-3590, Belgium; 3 Department of Computational Biology, Research and Innovation Center Fondazione Edmund MachSan Michele all'Adige, 38010, Trento (TN), Italy

**Keywords:** ECF sigma factor, EcfG, general stress response, PhyR, TcrX, *α*-proteobacteria

## Abstract

A core component of the *α*-proteobacterial general stress response (GSR) is the extracytoplasmic function (ECF) sigma factor EcfG, exclusively present in this taxonomic class. Half of the completed *α*-proteobacterial genome sequences contain two or more copies of genes encoding σ^EcfG^-like sigma factors, with the primary copy typically located adjacent to genes coding for a cognate anti-sigma factor (NepR) and two-component response regulator (PhyR). So far, the widespread occurrence of additional, non-canonical σ^EcfG^ copies has not satisfactorily been explained. This study explores the hierarchical relation between *Rhizobium etli* σ^EcfG1^ and σ^EcfG2^, canonical and non-canonical σ^EcfG^ proteins, respectively. Contrary to reports in other species, we find that σ^EcfG1^ and σ^EcfG2^ act in parallel, as nodes of a complex regulatory network, rather than in series, as elements of a linear regulatory cascade. We demonstrate that both sigma factors control unique yet also shared target genes, corroborating phenotypic evidence. σ^EcfG1^ drives expression of *rpoH2*, explaining the increased heat sensitivity of an *ecfG1* mutant, while *katG* is under control of σ^EcfG2^, accounting for reduced oxidative stress resistance of an *ecfG2* mutant. We also identify non-coding RNA genes as novel σ^EcfG^ targets. We propose a modified model for GSR regulation in *R. etli*, in which σ^EcfG1^ and σ^EcfG2^ function largely independently. Based on a phylogenetic analysis and considering the prevalence of *α*-proteobacterial genomes with multiple σ^EcfG^ copies, this model may also be applicable to numerous other species.

## Introduction

The general stress response (GSR) results in multiple stress resistance in stationary phase cells, allowing bacteria to survive adverse conditions. In *Escherichia coli* and many other proteobacteria, this stress response is controlled by the alternative sigma factor RpoS. Remarkably, members of the monophyletic class of *α*-proteobacteria lack an RpoS homologue. Rather, *α*-proteobacteria utilize a specific extracytoplasmic function (ECF) sigma factor, σ^EcfG^, that is exclusively present in this taxonomic class (Staron et al. 2009) and a unique response regulator, PhyR, composed of an N-terminal sigma factor-like domain and a C-terminal receiver domain. In the absence of stress, activity of σ^EcfG^ is restricted by an anti-sigma factor, NepR. Upon phosphorylation of PhyR, its N-terminal domain acts as a docking interface for NepR, thereby titrating it away from σ^EcfG^ and releasing the sigma factor to recruit RNA polymerase and to initiate transcription of σ^EcfG^-specific target genes. Studies in various *α*-proteobacteria support this partner-switching model (Gourion et al. 2008, 2009; Francez-Charlot et al. 2009; Bastiat et al. 2010; Kaczmarczyk et al. 2011; Lourenço et al. 2011; Abromaitis and Koehler 2013; Kim et al. 2013; Kulkarni et al. 2013) and, more recently, have provided a structural basis for the underlying protein-protein interactions (Campagne et al. 2012; Herrou et al. 2012). Homologues of σ^EcfG^, NepR, and PhyR are found in essentially all free-living *α*-proteobacteria but are absent in other classes. Interestingly, comparative genomic analyses revealed that about half of the completely sequenced genomes contain two or more copies of genes encoding σ^EcfG^-like sigma factors, while there is generally only one pair of PhyR and NepR homologues present (Staron and Mascher 2010). The widespread occurrence of genomes encoding multiple σ^EcfG^ proteins suggests an important selective advantage over having only a single copy. The exact function of these supplemental sigma factors, however, remains unclear, as research has so far mainly focused on the function of the primary σ^EcfG^ sigma factor, canonically located in the genomic vicinity of *phyR* and *nepR*.

*Rhizobium etli* is a soil-dwelling member of the *α*-proteobacteria, capable of infecting the roots of its leguminous host plant *Phaseolus vulgaris*, the common bean plant, in order to establish a nitrogen-fixing symbiosis. We previously studied the role of the alarmone (p)ppGpp in *R. etli* CNPAF512, recently reclassified as *Rhizobium phaseoli* (Lopez-Guerrero et al. 2012), and *R. etli* CFN42. (p)ppGpp, the effector molecule of the stringent response, is a widespread global regulatory system activated under unfavorable growth conditions (Braeken et al. 2006). Mutants unable to produce (p)ppGpp show severe defects in multiple stress resistance during free-living growth and symbiosis (Moris et al. 2005; Braeken et al. 2008). Based on a genome-wide transcriptome analysis, stress response regulators involved in the (p)ppGpp-dependent response were identified (Vercruysse et al. 2011), including σ^EcfG1^/RpoE4 and σ^EcfG2^/PF00052, the *R. etli* CFN42 members of the σ^EcfG^ group of sigma factors. Neither of the *R. etli* σ^EcfG^ proteins appear to play a major role in symbiotic nitrogen fixation (A. Jans, M. Vercruysse, M. Fauvart, and J. Michiels, unpubl. data), but rather participate in stress resistance. Interestingly, an *ecfG1* mutant primarily displays increased sensitivity to heat stress, while an *ecfG2* mutant is specifically sensitive to oxidative stress. An *ecfG1-ecfG2* double mutant exhibits even more pronounced stress susceptibility than either single mutant. These observations are at odds with a recently proposed model for the GSR in *Caulobacter crescentus*, in which σ^EcfG1^ functions as master regulator and exerts complete control over σ^EcfG2^, the latter merely amplifying the expression of a small subset of σ^EcfG1^ target genes (Lourenço et al. 2011).

In this study, we attempt to resolve this matter by charting the regulatory network that encompasses *R. etli* σ^EcfG1^ and σ^EcfG2^. We demonstrate σ^EcfG1^-independent expression of σ^EcfG2^ and preferential recognition by each sigma factor of the own promoter sequence. Furthermore, we show that both sigma factors control unique yet also shared target genes, corroborating phenotypic evidence. We also identify non-coding RNAs (ncRNAs) as novel σ^EcfG^ targets and show that expression of at least one of these ncRNAs is under direct σ^EcfG^ control. Considering the widespread existence of *α*-proteobacteria with multiple σ^EcfG^ copies, these results may contribute to a more broadly applicable model for GSR regulation.

## Material and Methods

### Bacterial strains, media and growth conditions

Strains and plasmids used in this study are listed in Table S1. *R. etli* strains were grown as described previously (Michiels et al. 1994). *E. coli* strains were cultured at 37°C in lysogeny broth (LB). When appropriate, following antibiotics (Sigma-Aldrich, St. Louis, MO) were supplied: ampicillin (100 μg mL^−1^); gentamicin (30 μg mL^−1^); kanamycin (40 μg mL^−1^); nalidixic acid (15 μg mL^−1^); neomycin (35 μg mL^−1^); spectinomycin (50 μg mL^−1^ for *E. coli* or 25 μg mL^−1^ for *R. etli*) and tetracycline (10 μg mL^−1^ for *E. coli* or 1 μg mL^−1^ for *R. etli*). Arabinose (VWR, Radnor, PA) was dissolved (20% w/v) in distilled water and filter sterilized before use.

### Controlled expression of *ecfG1* and *ecfG2*

The *ecfG1* gene was amplified by polymerase chain reaction (PCR) from *R. etli* CFN42 genomic DNA using primers SPI 3050 and SPI 3051 (Table S2). Following digestion with XhoI and HindIII, the 0.6-kb fragment was cloned into pBAD/HisA (Invitrogen, Carlsbad, CA), resulting in pCMPG13516. Similarly, the *ecfG2* gene was amplified using SPI 4317 and SPI 4318 and after digestion with *Xho*I and *Hind*III, the 0.5-kb fragment was cloned into pBAD/HisA, resulting in pCMPG13517. Constructs were confirmed by sequencing and expression following induction by arabinose was verified by western blotting and hybridization using anti-His_6_ antibodies. For both constructs, protein expression levels were comparable.

### Mutant construction

A *phyRtcrY* mutant (CMPG13304) was constructed by first amplifying a 3.5-kb fragment using Platinum *Pfx* DNA polymerase (Invitrogen) and primers SPI 0482 and SPI 0483, which carried *Not*I recognition sites at their 5′ ends. The resulting fragment was cloned into pCR4Blunt-TOPO (Invitrogen) and confirmed by sequencing. A 1.5-kb fragment internal to *phyRtcrY* was removed using *Sac*I and *Nsi*I and replaced by a spectinomycin resistance cassette isolated from pHP45ΩSp. A 4-kb *Not*I-fragment from the resulting construct was cloned into the *Not*I site of pJQ200-uc1, giving rise to pCMPG13518. Finally, this plasmid was used for site-directed mutagenesis of *phyRtcrY* following triparental conjugation as described by D'Hooghe et al. (1995). The obtained mutants were verified by Southern blot hybridization as optimized by D'Hooghe et al. (1997).

Primers SPI 0484 and SPI 0485, carrying *Not*I recognition sites at their 5′ ends, were used to amplify the 2.0-kb *ecfG1* region from *R. etli* CFN42 genomic DNA by PCR using *Pfx* DNA polymerase. The resulting fragment was cloned into pCR4Blunt-TOPO (Invitrogen) confirmed by sequencing, and a Km^R^-cassette, obtained from pHP45ΩKm, was inserted in the *Nsi*I site of *ecfG1*. The corresponding *Not*I-fragment was removed and cloned into the suicide plasmid pJQ200-uc1, resulting in pCMPG13519. This pJQ200-uc1 construct was again used for site-directed mutagenesis and obtained mutants were verified by Southern blot hybridization.

### Construction of transcriptional gusA fusions

Transcriptional fusions between the putative promoter regions of *phyR*, *ecfG1*, *ecfG2,* and ReC64 and a promoterless *gusA* reporter gene were constructed as follows. The different regions were amplified from *R. etli* CFN42 genomic DNA by PCR with *Pfx* DNA polymerase. Following primers were used: *phyR*-*ecfG1*: SPI 1422/1423; ReC64: SPI 2538/3231 and *ecfG2*: SPI 7864/8009. The corresponding fragments were cloned into pCR4Blunt-TOPO, confirmed by sequencing and subcloned into pFAJ1703, resulting in pCMPG13512 to pCMPG13515.

### Determination of *β*-glucuronidase activity

Quantitative analysis of GusA activity was carried out as described previously (Michiels et al. 1998). In short, *R. etli* cells were grown at 30°C in TY medium, while monitoring the optical density (OD) of the culture. Samples were taken at OD_595_ = 0.85, representing stationary phase. *E. coli* cells were grown in LB medium and samples were taken at OD_595_ = 0.5. GusA expression assays were carried out using *p*-nitrophenyl-*β*-d-glucuronide as a substrate for *β*-glucuronidase. Experiments were carried out at least in triplicate and confirmed in independent repeats.

### RNA isolation and cDNA synthesis

Total RNA was isolated using a previously optimized protocol (Vercruysse et al. 2010). In short, the RNA content of 50 mL bacterial culture in early stationary phase, grown in rich medium without treatment was stabilized using a phenol:ethanol (5:95) solution. Cells were flash frozen in liquid nitrogen and stored at −80°C. Total RNA was extracted using the TRIzol Plus RNA Purification System (Invitrogen). DNA contamination was removed by two treatments with 2 μL TURBO DNase (Ambion, Austin, TX) and afterwards verified by PCR (30 cycles). RNA integrity was analyzed using Experion RNA StdSens Chips (Bio-Rad, Hercules, CA), RNA quantity and purity were assessed using a NanoDrop ND-1000. For RT-qPCR analysis, 1 μg of total RNA was reverse transcribed to single-stranded cDNA using the SuperScript VILO cDNA Synthesis Kit (Invitrogen) according to the manufacturer's protocol. For microarray detection, double-stranded cDNA was synthesized using random decamers (Ambion) and the SuperScript Double-Stranded cDNA Synthesis Kit (Invitrogen) according to the manufacturer's protocol.

### High-density microarray design and data preprocessing

A whole-genome tiling array covering the entire *R. etli* genome sequence (6.5 Mbp in total) was designed by NimbleGen Systems, Inc. (Madison, WI) with ∼385.000 60*mer* probes having an average start-to-start spacing of 13 bp. Samples were hybridized and scanned by NimbleGen. Submission of the data to the National Center for Biotechnology Information GEO database is in progress.

Data preprocessing was performed as described previously (Vercruysse et al. 2011). Briefly, a robust estimation of the noise in the expression data was carried out to determine the significant levels of gene expression. Subsequently, the absolute expression ratio of all genes was determined, using the wild-type strain as a reference. If this ratio were greater than or equal to 2 (log_2_ ≥ 1), the genes were considered to be differentially expressed.

### Sequence analysis

Sequences −350 bp to +10 bp (relative to the predicted start codon) upstream of the identified target genes were screened for the presence of overrepresented motifs using the MEME program of the MEME SUITE platform (Bailey et al. 2009) with a motif width between 25 and 30. Sequence retrieval and motif matching was done using the retrieve sequence and matrix-scan programs, respectively, from the RSAT web site (Foreman et al. 2012).

For phylogenetic tree construction, EcfG protein sequences from selected members of the Rhizobiales, Caulobacterales and Sphingomonadales were retrieved from GenBank (NCBI). Further analysis was carried out out using MEGA5 (Crossman et al. 2008) as described previously (Fauvart et al. 2009).

### RT-qPCR

Expression levels were determined by reverse transcription quantitative real-time PCR (RT-qPCR) using the StepOnePlus System and SYBR Green, as described previously (Vercruysse et al. 2010). Primers were designed using Primer Express 3.0 (Table S2). Secondary structures and dimer formation were checked with Oligoanalyzer 3.1. In order to ensure that there was no background contamination, a negative control was included in each run. All reactions were performed in triplicate and carried out in fast optical 96-well reaction plates (MicroAmp, Applied Biosystems, Foster City, CA). Data were analyzed using StepOne Software v2.2. RNA isolated from wild-type *E. coli* or *R. etli* was used as calibrator condition, 16S rRNA was used as a reference gene. Relative gene expression was calculated using the Pfaffl method (Pfaffl 2001).

### 5′ RACE

5′ RACE was performed as described previously (Vercruysse et al. 2010). Sequences of the gene-specific inner and outer primers are listed in Table S2.

## Result and Discussion

### Many *α*-proteobacteria encode multiple *ecfG* copies

One of the core components of the *α*-proteobacterial partner-switching model controlling the GSR is a sigma factor belonging to the ECF15/σ^EcfG^ group (Staron et al. 2009). Interestingly, a survey of the Microbial Signal Transduction (MiST) database (Ulrich and Zhulin 2010) revealed that about half of the completely sequenced genomes of *α*-proteobacteria contain multiple sigma factors belonging to this group (Table S3). *R. etli* CFN42 carries two genes encoding σ^EcfG^-type sigma factors: the chromosome-encoded *rpoE4* (CH03273) and the plasmid-borne PF00052. An anti-sigma factor coding gene (CH03274) is located upstream of *rpoE4* and it was previously reported that both genes form an operon (Martinez-Salazar et al. 2009a). Genes encoding a two-component regulatory system, composed of a response regulator annotated as TcrX (two-component regulator; locus CH03275), a PhyR orthologue, and a sensor histidine kinase TcrY (CH03276), are found upstream and divergently oriented from this transcriptional unit. No genes encoding a response regulator nor an anti-sigma factor are found in the genomic vicinity of PF00052. A revised ECF sigma factor nomenclature was recently proposed for ECF15/σ^EcfG^-like sigma factors (Staron et al. 2009). Accordingly, we will henceforth refer to the canonical *R. etli* RpoE4 as σ^EcfG1^ and to the non-canonical PF00052 as σ^EcfG2^. For reasons of clarity and uniformity, we propose to rename *R. etli* TcrX to PhyR.

### Expression of *ecfG1*, *ecfG2*, and *phyR*

To analyze the regulatory hierarchy of the *R. etli* GSR, the expression levels of *phyR*, *ecfG1*, and *ecfG2* were quantitatively evaluated using promoter fusions to a promoterless *gusA* reporter gene in wild-type *R. etli* and mutant strains *ΔphyRtcrY*, Δ*ecfG1*, Δ*ecfG2*, and Δ*ecfG1*Δ*ecfG2*. Moreover, to distinguish between direct and indirect effects, expression levels of the different promoter fusions were also measured following controlled expression of σ^EcfG1^ or σ^EcfG2^ in the heterologous host *E. coli*.

**Figure 1:**
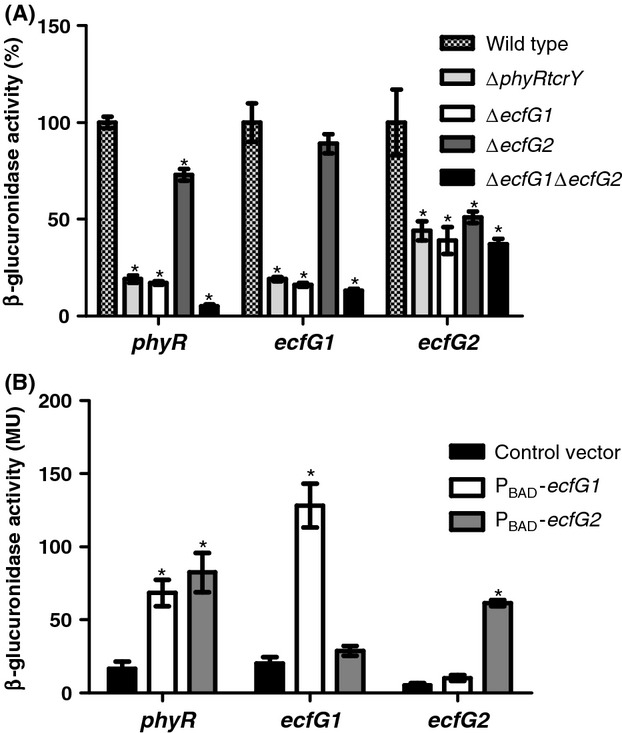
Expression of *phyR*-*gusA*, *ecfG1*-*gusA* and *ecfG2-gusA* transcriptional reporter fusions. (A) GusA expression in wild-type *R. etli* CFN42, Δ*phyRtcrY*, Δ*ecfG1,* Δ*ecfG2,* and Δ*ecfG1*Δ*ecfG2* backgrounds. Expression levels are shown relative to expression in the wild type and are the means of three biological replicates with bars representing the standard deviation. Statistically significant differences in expression compared to expression in the wild-type background are marked with an asterisk (*P* < 0.05). (B) GusA expression in the heterologous host *E. coli*. Plasmid-borne copies of *R. etli ecfG1* and *ecfG2* were expressed under control of an arabinose-inducible promoter**.** Expression in the presence of the empty plasmid was included as negative control. Expression levels are shown in Miller units and are the means of six biological replicates with bars representing the standard deviation. Statistically significant differences in expression compared to the negative control are marked with an asterisk (*P* < 0.05).

In line with previous results (Martinez-Salazar et al. 2009a), *phyR* expression is severely reduced in a Δ*ecfG1* strain (Fig. 1A). Surprisingly, there is also a modest but significant (*P* < 0.05) decrease in a Δ*ecfG2* strain and, consistently, nearly no detectable expression in a Δ*ecfG1*Δ*ecfG2* strain*,* suggesting that, like σ^EcfG1^, σ^EcfG2^ positively affects *phyR* expression. This observation is confirmed by results in *E. coli*, in which the *phyR* promoter is recognized by both σ^EcfG1^ and σ^EcfG2^ (Fig. 1B).

Consistent with the current GSR model (Staron and Mascher 2010), there is a clear drop in *ecfG1* expression in a *phyRtcrY* mutant (Fig. 1A). There is no significant (*P* < 0.05) reduction in *ecfG1* expression in a *R. etli ecfG2* mutant (Fig. 1A), suggesting complete σ^EcfG2^-independence. On the other hand, *ecfG2* expression is reduced to about half of wild-type level in the Δ*ecfG1* mutant, indicating partial σ^EcfG1^-dependence of *ecfG2* expression. Importantly, however, this also strongly suggests a significant level of σ^EcfG2^ expression independent of PhyR and σ^EcfG1^.

Expression of *ecfG1* is autoregulated, as expression of an *ecfG1* promoter fusion is almost abolished in *R. etli* strains lacking this sigma factor (Fig. 1A) and is strongly induced in the presence of excess σ^EcfG1^ (Fig. 1B). Likewise, expression of an *ecfG2* promoter fusion is significantly (*P* < 0.05) induced when σ^EcfG2^ is overexpressed, indicating *ecfG2* expression is also autoregulated. Additionally, the *ecfG1* promoter fusion is not significantly (*P* < 0.05) induced in the heterologous system overexpressing σ^EcfG2^ (Fig. 1B), confirming that *ecfG1* expression is σ^EcfG2^-independent. Moreover, σ^EcfG1^ overexpression does not directly stimulate *ecfG2* expression, thus demonstrating for each σ^EcfG^ copy preferential recognition of its own promoter, at least as part of the *E. coli* RNA polymerase complex.

The complete σ^EcfG2^-independence of *ecfG1* expression and partial σ^EcfG1^-dependence of *ecfG2* expression suggest that PhyR and σ^EcfG1^ constitute a core module of the GSR while σ^EcfG2^, on the other hand, seems to function as an accessory module. Significant expression of *ecfG2* in the absence of PhyR and σ^EcfG1^ supports the notion that σ^EcfG2^ is also part of a stress resistance pathway operating independently of the σ^EcfG1^-mediated GSR. Strikingly, σ^EcfG1^ and σ^EcfG2^ not only appear to differ in upstream control, but downstream as well, illustrated by the observation that both sigma factors recognize the *phyR* promoter, while preferentially stimulating their own expression over that of their respective σ^EcfG^ paralogue. This contrasts sharply with the recently described model for dual σ^EcfG^ control in *C*. *crescentus*, where *ecfG2* (*sigU*) expression is completely abolished in an *ecfG1* (*sigT*) mutant and a more modest role was proposed for σ^EcfG2^, that is to amplify the σ^EcfG1^-mediated response (Lourenço et al. 2011).

### σ^EcfG1^ and σ^EcfG2^ regulon delineation

We next examined whether the observed differences in upstream and downstream control also result in distinct regulons for σ^EcfG1^ and σ^EcfG2^. To this end, comparative transcriptome analyses were carried out with the parental strain and mutants in either *ecfG1*, *ecfG2* or both genes combined. Based on previously optimized conditions, total RNA was obtained from early stationary phase cultures and hybridized to a custom-design genome-wide tiling array (Vercruysse et al. 2010). The microarray data were validated by analyzing the expression levels of 13 arbitrarily selected genes using reverse transcription quantitative polymerase chain reaction (Fig. S1).

**Figure 2:**
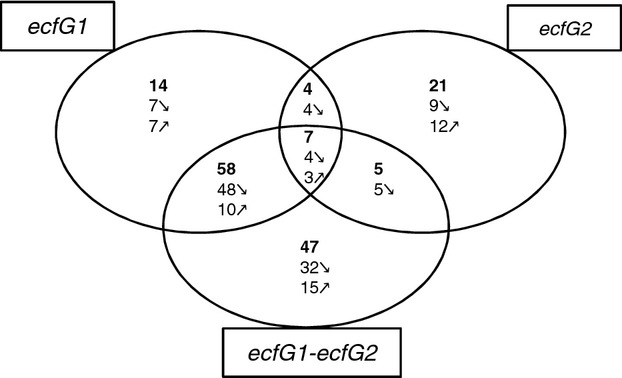
σ^EcfG1^-, σ^EcfG2^- and σ^EcfG1^-σ^EcfG2^-dependent gene expression. Venn diagram of all differentially expressed genes in *ΔecfG1*, *ΔecfG2*, and *ΔecfG1ΔecfG2* mutants compared to the wild-type strain *R. etli* CFN42. Upward- and downward-oriented arrows indicate gene induction and repression, respectively.

Overall, 83 genes are differentially expressed in the *ecfG1* mutant, 37 in the *ecfG2* mutant and 117 in the Δ*ecfG1*Δ*ecfG2* double mutant (Fig. 2). Interestingly, overlap between the σ^EcfG1^ and σ^EcfG2^ regulons is limited, with only 11 genes in common, whereas the *ecfG1* mutant and the Δ*ecfG1*Δ*ecfG2* double mutant share the majority of differentially expressed genes, 65 in total. These results demonstrate that σ^EcfG1^ and σ^EcfG2^ control the expression of a partially distinct set of target genes, with a large number of shared target genes requiring the presence of either σ^EcfG1^ or σ^EcfG2^, a few needing both, and with a limited number of unique targets for each sigma factor.

The observation that *R. etli* σ^EcfG1^ and σ^EcfG2^ each control unique target genes is consistent with our previous finding that both sigma factors recognize specific promoter sequences (Fig. 1) and that *ecfG1* and *ecfG2* mutants display distinct phenotypes when exposed to heat shock or oxidative stress (Vercruysse et al. 2011). An *ecfG1* mutant has a decreased viability after heat shock, while an *ecfG2* mutant exhibits a more severe oxidative stress phenotype than an *ecfG1* mutant. Additionally, the even more pronounced stress susceptibility of a Δ*ecfG1*Δ*ecfG2* double mutant can be explained by the relatively large number of genes that is differentially expressed only in the absence of both σ^EcfG1^ and σ^EcfG2^.

The presence of *rpoH2* in the σ^EcfG1^ regulon can account for the reduced viability we observed of an *ecfG1* mutant when exposed to elevated temperatures, as *R. etli rpoH2* contributes to heat stress resistance (Martinez-Salazar et al. 2009b). Decreased expression in an *ecfG2* mutant of *katG*, encoding a catalase, may explain the increased sensitivity to oxidative stress of an *ecfG2* mutant (Vercruysse et al. 2011), as previous studies have found KatG to be important in *R. etli* oxidative stress resistance (Vargas Mdel et al. 2003; Dombrecht et al. 2005; Garcia-de Los Santos et al. 2008).

### σ^EcfG1^ and σ^EcfG2^ promoter motifs

In order to discriminate between direct and indirect targets of σ^EcfG1^ and σ^EcfG2^ in their respective regulons, a motif search was performed. Analysis of the promoter regions of σ^EcfG1^-regulated genes identified a GGAAC-N16-CGTT sequence, perfectly matching the motif previously reported for *R. etli* σ^EcfG1^ (Martinez-Salazar et al. 2009a). Of the 56 putative transcriptional units downregulated in an *ecfG1* mutant, 19 are preceded by the motif, indicative of direct regulation by σ^EcfG1^. A search in the downregulated genes of the Δ*ecfG1*Δ*ecfG2* double mutant resulted in exactly the same motif, preceding 22 out of 82 putative transcriptional units (Table S4). This is not surprising considering the large overlap between both gene sets (Fig. 2). However, a search for an overrepresented motif in the promoter sequences of the σ^EcfG2^-regulated genes did not return any hits. This is possibly due to the limited size of the dataset, and may be improved upon future studies by using conditions that more specifically induce *ecfG2* expression, or alternatively, by ectopically overexpressing σ^EcfG2^ in a Δ*ecfG1*Δ*ecfG2* background as was previously done for *C. crescentus* (Lourenço et al. 2011). Surprisingly, of the 19 putative transcriptional units downregulated in an *ecfG2* mutant, only 1 has a promoter sequence that matches the σ^EcfG1^ consensus motif (Table S4).

### (p)ppGpp-dependency of σ^EcfG1^ and σ^EcfG2^ target genes

(p)ppGpp, a hyperphosphorylated guanosine nucleotide, was originally characterized as the effector molecule of the stringent response to nutritional stress. However, it has since become clear that the function of the alarmone is more versatile and that (p)ppGpp induces profound physiological alterations in response to unfavorable growth conditions by regulating a global reprogramming of gene expression as well as translation and DNA replication (Braeken et al. 2006; Abromaitis and Koehler 2013).

In a previous study, we found that expression of both *ecfG1* and *ecfG2* is alarmone-dependent (Vercruysse et al. 2011), suggesting that (p)ppGpp might be an important input signal to switch on the σ^EcfG^-dependent GSR in *R. etli*. We therefore explored to what extent the expression of genes present in the σ^EcfG1^ and σ^EcfG2^ regulons is also (p)ppGpp-dependent. Comparison of the differentially expressed genes in a *rsh* mutant, unable to produce (p)ppGpp, and the *ecfG1* mutant, identified 33 genes (40%) in the σ^EcfG1^ regulon whose expression is also *rsh*-dependent. The σ^EcfG2^ and *Rsh* regulons share 7 genes (19%), while a Δ*ecfG1*Δ*ecfG2* double mutant and an *rsh* mutant have 41 genes in common (35%). Moreover, if we take into account only those genes preceded by an σ^EcfG1^ consensus motif, expression of 68% (13/19) of the genes in the σ^EcfG1^ regulon and 73% (16/22) of the genes in the σ^EcfG1^-σ^EcfG2^ regulon is alarmone-dependent, confirming the stringent response as an important driver of σ^EcfG^ expression in *R. etli*. This is similar to the situation in *E. coli*, where (p)ppGpp is a major signal responsible for the induction of the RpoS-mediated GSR. Besides a positive regulation of *rpoS* transcription and translation, (p)ppGpp enables RpoS to compete with the housekeeping sigma factor (RpoD) for binding RNA polymerase, thereby shifting gene expression from a predominantly RpoD-regulated expression during exponential growth to an RpoS-regulated expression in stationary phase (Jishage et al. 2002; Battesti et al. 2011).

### σ^EcfG1^ and σ^EcfG2^ control expression of non-coding RNAs

Previously, 28 ncRNAs were identified as positively regulated by (p)ppGpp in *R. etli* (Vercruysse et al. 2011), suggesting that ncRNAs may be involved in *R. etli* stress resistance. We therefore quantified ncRNA expression in the *ecfG1* and *ecfG2* mutants. A total of 14 ncRNAs was found to be differentially expressed in at least one of the mutant stains, 6 of which are downregulated and 8 upregulated (Fig. S2). Half of them (7/14) are also regulated by the alarmone (p)ppGpp. Expression of 4 ncRNAs is σ^EcfG1^-dependent, 2 are σ^EcfG2^-dependent and 8 display a combined σ^EcfG1^-σ^EcfG2^ dependency. Interestingly, while there is a considerable overlap between the σ^EcfG1^- and σ^EcfG1^-σ^EcfG2^-dependent ncRNAs, the regulons of *ecfG1* and *ecfG2* mutants have only 2 ncRNAs in common, further corroborating our finding that both sigma factors control distinct regulons.

**Figure 3:**
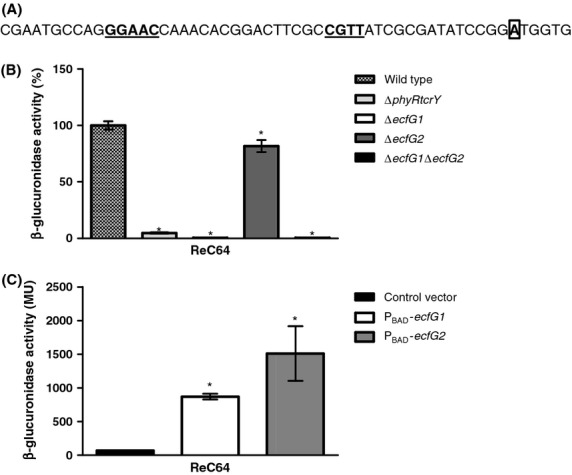
Transcriptional control of ncRNA ReC64 expression. (A) Identification of the transcription initiation site of ReC64 by 5′ RACE. The σ^EcfG1^ consensus motif present in the putative ReC64 promoter sequence is shown in boldface and underlined. The experimentally determined transcription initiation site is shown in boldface and is boxed. 5′ RACE was performed as described previously (Vercruysse et al. 2010). (B) Expression of the ReC64-*gusA* transcriptional promoter fusion in wild-type *R. etli* CFN42, Δ*phyRtcrY*, Δ*ecfG1,* Δ*ecfG2*, and Δ*ecfG1*Δ*ecfG2*. GusA expression levels are shown relative to expression in wild-type *R. etli* CFN42 and are the means of three biological replicates with bars representing the standard deviation. Statistically significant differences in expression compared to expression in the wild-type background are marked with an asterisk (*P* < 0.05). (C) Expression of the ReC64-*gusA* transcriptional promoter fusion in the heterologous host *E. coli*. Plasmid-borne copies of *R. etli ecfG1* and *ecfG2* were expressed under control of an arabinose-inducible promoter**.** Expression in the presence of the empty plasmid was included as negative control. Expression levels are shown in Miller units and are the means of six biological replicates with bars representing the standard deviation. Statistically significant differences in expression compared to the negative control are marked with an asterisk (*P* < 0.05).

Five of the six downregulated ncRNAs are expressed in an σ^EcfG1^-dependent manner, including the highly conserved ncRNAs ReC55 (RNase P) and ReC70 (6S RNA) and ReC64, The latter is located in the intergenic region downstream of the *phyRtcrY* locus and can therefore be considered as a transencoded ncRNA. The presence of ReC64 is intriguing, as it is the only downregulated ncRNA that is preceded by the σ^EcfG1^ consensus promoter motif (Fig. 3A). Moreover, it is located in the highly conserved *ecfG1*-*phyR* genomic region and based on the microarray data (Table S4), its expression is significantly (*P* < 0.01) reduced in all three mutants. ReC64 was first identified in a genome-wide detection of predicted ncRNAs (Vercruysse et al. 2010). The ncRNA is conserved in *R. etli* CIAT 652 and *R. leguminosarum* biovar *viciae* 3841 and its expression and transcript length (88 bp) were previously confirmed by Northern analysis (Vercruysse et al. 2010). We here determined the transcription initiation site of ReC64, located downstream of the histidine kinase gene *tcrY*, by 5′ RACE and found it in agreement with the expected transcription initiation site, based on the position of the σ^EcfG1^ consensus promoter motif (Fig. 3A).

To further elucidate the transcriptional regulation of the ncRNA, ReC64 expression levels were evaluated in different mutant backgrounds using a *gusA* reporter fusion as described above for *ecfG1* and *ecfG2*. As shown in Figure 3B, ReC64 expression is significantly (*P* < 0.05) reduced in all mutant strains. While the expression level in an *ecfG2* mutant is still 82% of that in the wild-type background, expression is abolished in *ecfG1* and Δ*ecfG1*Δ*ecfG2* mutants, suggesting that expression of ReC64 is predominantly σ^EcfG1^-dependent. These findings are in good agreement with our microarray expression data. Expression of the promoter fusion in *E. coli* is strongly activated in the presence of σ^EcfG1^ as well as of σ^EcfG2^ (Fig. 3C), supporting direct regulation of ReC64 by σ^EcfG^.

The presence of ncRNAs in the σ^EcfG1^ and σ^EcfG2^ regulons in *R. etli* is reminiscent of the situation in several gamma-proteobacteria, where the presence of ncRNAs in the RpoS regulon has been described earlier. In *Salmonella enterica* serovar Typhimurium, IsrE is involved in the response to iron starvation (Padalon-Brauch et al. 2008); in *E. coli*, GadY is responsible for the regulation of acid response genes (Opdyke et al. 2004); and SdsR, widely conserved in Enterobacteriaceae, controls the synthesis of the major porin OmpD (Frohlich et al. 2012). Whether ncRNAs are also part of the GSR regulon in other *α*-proteobacteria, and whether they play any role in stress resistance, remains to be investigated.

### Modified model for regulation of the general stress response

**Figure 4:**
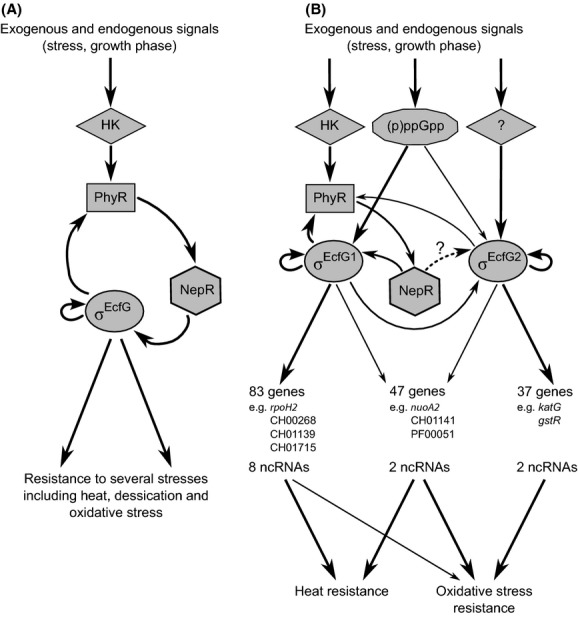
Schematic diagrams of the *α*-proteobacterial GSR network involving one or multiple σ^EcfG^ copies. (A) Generalized model for the regulatory network involving one σ^EcfG^. Exogenous (e.g., stress) and endogenous (e.g., growth phase) signals can switch on the GSR. Activation takes place through a sensory histidine kinase (HK) that modulates PhyR. Activated PhyR competes for NepR binding, alleviating σ^EcfG^ sequestration by NepR. σ^EcfG^ target genes are involved in resistance against heat, dessication and oxidative stress. (B) Proposed model for GSR regulation by multiple σ^EcfG^ copies, for example, in *R. etli* by σ^EcfG1^ and σ^EcfG2^. Exogenous (e.g., stress) and endogenous (e.g., growth phase) signals switch on the GSR, directly or through (p)ppGpp signaling. For σ^EcfG1^, direct activation takes place through a sensory histidine kinase (HK) that modulates PhyR. Activated PhyR competes for NepR binding, alleviating σ^EcfG1^ sequestration by NepR. For σ^EcfG2^, direct activation can occur in both a HK/PhyR/NepR/σ^EcfG1^-dependent and independent manner. σ^EcfG1^ and σ^EcfG2^ control distinct regulons, including protein-coding genes and ncRNAs, but also display some functional redundancy. σ^EcfG1^ target genes are involved in resistance against oxidative and heat stress while σ^EcfG2^ target genes provide protection against oxidative stress. The thickness of the arrows indicates the impact on regulation of each component. See text for further details.

On the basis of the results obtained in this study, we propose a modified model for the regulation of the *R. etli* GSR by σ^EcfG1^ and σ^EcfG2^ (see Fig. 4). As the presence of multiple σ^EcfG^ copies is widespread in genomes of *α*-proteobacteria, the model may also be of predictive value in numerous additional bacterial species.

Our results confirm the generally accepted position of PhyR as hierarchically superior to σ^EcfG1^ and σ^EcfG2^. However, significant σ^EcfG2^ expression in the absence of PhyR suggests regulatory inputs independent of the PhyR/σ^EcfG1^-mediated GSR. Transcriptome analyses reveal that there is only limited overlap between the σ^EcfG1^ and σ^EcfG2^ regulons. Moreover, both sigma factors preferentially recognize their own promoter sequence, as demonstrated by promoter activity analysis in the presence of controlled expression of σ^EcfG1^ or σ^EcfG2^. Taken together, these observations suggest a model in which both sigma factors act largely independently. Regulon analysis in a Δ*ecfG1*Δ*ecfG2* double mutant, however, suggests that both sigma factors are, partly, functionally redundant, a proposition corroborated by the observation that the *phyR* promoter region is recognized by both σ^EcfG1^ and σ^EcfG2^ in the heterologous host *E. coli*. Therefore, it is likely that regulation of the GSR is a complex process and that other factors, such as the expression levels of the respective sigma factors or the nature of the stress triggering the response, might affect functioning of σ^EcfG1^ and σ^EcfG2^. Restriction of σ^EcfG^ activity by the anti-sigma factor NepR in the absence of stress is a common feature of the *α*-proteobacterial GSR. Consistently, control of *R. etli* σ^EcfG1^ activity by NepR has been previously described (Martinez-Salazar et al. 2009a). Whether σ^EcfG2^ activity is also regulated through interaction with NepR is currently unclear. However, studies in *C. crescentus* and *Sphingomonas* sp. Fr1 revealed no interaction between NepR and σ^EcfG2^ (Kaczmarczyk et al. 2011; Lourenço et al. 2011).

### Phylogenetic analysis

**Figure 5:**
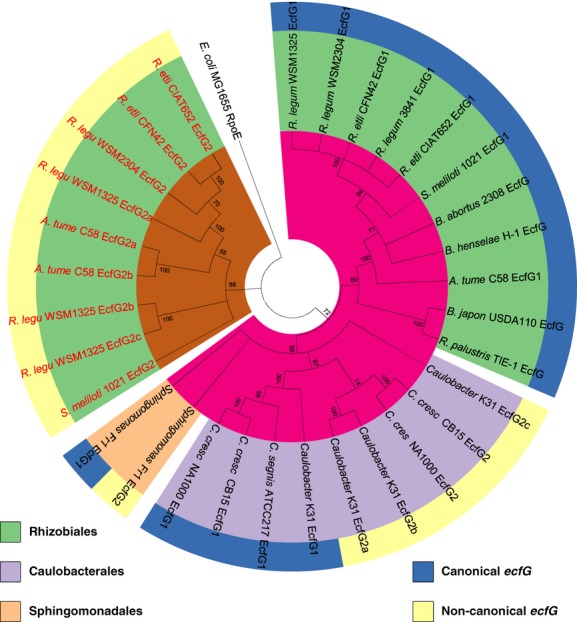
Neighbor-joining phylogenetic tree of σ^EcfG^-like sequences. Protein sequences of selected members of the Rhizobiales, Caulobacterales, and Sphingomonadales were aligned using RpoE of *E. coli* MG1655 as outgroup. Proteins encoded by chromosomally located genes are indicated in black, those encoded by plasmid-borne genes are indicated in red. Bootstrap values of 100 replicates are shown at the nodes for values >50. Two distinct subgroups (shown in pink and brown) can be observed. Essentially, the same tree topography was obtained using Maximum Likelihood and Minimal Evolution methods. σ^EcfG^ protein sequences were retrieved from GenBank (NCBI). Further analysis was carried out using MEGA5 (Crossman et al. 2008) as described previously (Fauvart et al. 2009).

Our experimental data suggest a model in which *R. etli* σ^EcfG2^ acts largely independently of σ^EcfG1^. This seems at odds with findings described for *C. crescentus* and *Sphingomonas* sp. Fr1 (Kaczmarczyk et al. 2011; Lourenço et al. 2011). A possible explanation may lie in the interrelatedness of the respective genes and their gene products: phylogenetic analysis shows that *R. etli* σ^EcfG1^, *C. crescentus* σ^EcfG1^ (SigT) and σ^EcfG2^ (SigU), and *Sphingomonas* sp. Fr1 σ^EcfG^ and σ^EcfG2^ cluster together more tightly than do *R. etli* σ^EcfG1^ and σ^EcfG2^ (see Fig. 5). Rather, they are part of distinct subgroups (shown in pink and brown, respectively), indicative of a relatively ancient duplication event dating back to a common ancestor of the Rhizobiales. It is not unlikely that the process leading to the considerable sequence divergence of *R. etli* σ^EcfG1^ and σ^EcfG2^, as compared to the σ^EcfG^ proteins of *Caulobacter* and *Sphingomonas*, was accompanied by diversification at both the functional and regulatory levels, as is apparent from our experimental data. Strikingly, members of the subgroup containing *R. etli* σ^EcfG1^ (shown in pink in Fig. 5) are all encoded by genes located on the chromosome. In contrast, all members of the *R. etli* σ^EcfG2^ subgroup correspond to plasmid-borne genes (shown in brown in Fig. 5). It has previously been suggested that chromosomes carry the “core genome” of a species, with well-conserved genes that are crucial for basic cell physiology, while plasmids represent the “accessory genome”, with adaptive genes that evolve more rapidly (Young et al. 2006; Crossman et al. 2008). This would explain why σ^EcfG1^ orthologues are virtually omnipresent, and why σ^EcfG2^ orthologues are not. In addition, it accounts for the observed functional, regulatory, and sequence divergence of the *R. etli* σ^EcfG2^ subgroup. Clearly, this matter warrants further investigation.

## Conclusions

In this study, we aimed to elaborate a model for *α*-proteobacterial GSR regulation by multiple σ^EcfG^ proteins. As half of the completely sequenced genomes of *α*-proteobacteria encode at least two σ^EcfG^ proteins, the implications of such a model are potentially far-reaching.

By determining the regulon of σ^EcfG1^ and σ^EcfG2^, the *R. etli* members of the σ^EcfG^ group of sigma factors, and examining the interplay between them, we demonstrated that σ^EcfG1^ and σ^EcfG2^ control, at least in part, distinct regulons, although some functional redundancy was observed as well. We identified the alarmone (p)ppGpp as an important upstream mediator of the GSR and discovered ncRNAs in the regulons of both sigma factors. Collectively, these results lead to a modified model for GSR regulation, in which σ^EcfG1^ and σ^EcfG2^ function largely independently. Together with the presented *in vivo* data, the *in silico* analysis of the phylogenetic relation of functionally characterized σ^EcfG^ proteins hints at a thus far unsuspected plasticity of the GSR network architecture in various lineages of *α*-proteobacteria. Our combined results pave the way for an in-depth study of these relations across the wealth of publicly available genome sequence data and are likely to have important evolutionary implications.

## Supporting Information

**Figure S1.** RT-qPCR validation of the microarray data. Expression of 13 genes was determined using RT-qPCR for wild type, Δ*ecfG1*, Δ*ecfG2*, and Δ*ecfG1*Δ*ecfG2*. The log_2_ transformed mean values of three replicates were used to report three different fold changes for each gene (*Y*-axis) compared to the respective microarray fold changes (*X*-axis). Black squares represent wild type versus Δ*ecfG1*; light gray diamonds wild type versus Δ*ecfG2* and dark gray dots wild type versus Δ*ecfG1*Δ*ecfG2*

**Figure S2.** σ^EcfG1^-, σ^EcfG2^- and σ^EcfG1^-σ^EcfG2^-dependent ncRNA expression. Venn diagram of all differentially expressed ncRNAs in Δ*ecfG1*, Δ*ecfG2*, and Δ*ecfG1*Δ*ecfG2* strains compared to the wild-type strain *R. etli* CFN42. Upward- and downward-oriented arrows indicate gene induction and repression, respectively.

**Table S1.** Bacterial strains and plasmids used in this study.

**Table S2.** Primers used in this study.

**Table S3.** Distribution of σ^EcfG^ sigma factors in completely sequenced α-proteobacterial genomes. Data retrieved from MiST database (http://mistdb.com; (Ulrich and Zhulin 2010) on 29 November 2012.

**Table S4.** The differentially expressed genes and ncRNAs in Δ*ecfG1*, Δ*ecfG2*, and Δ*ecfG1*Δ*ecfG2* compared to the wild type.
