# Supplementary figures and images for: Canonical and non-canonical EcfG sigma factors control the general stress response in Rhizobium etli
Source: Microbiologyopen. 2013 Oct 28;2(6):976–87. doi: 10.1002/mbo3.137 (PMC3892343; doi:10.1002/mbo3.137)

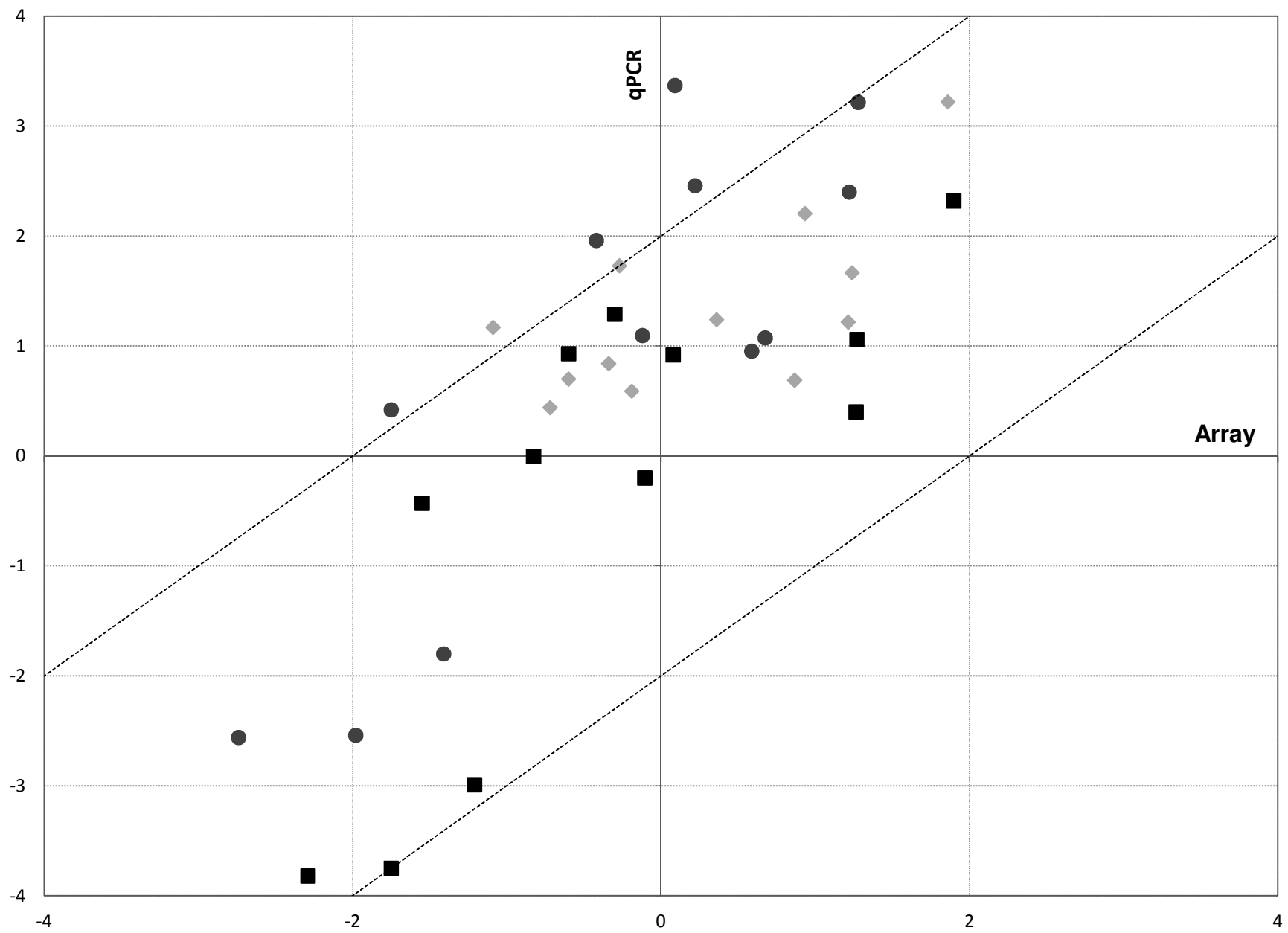

Supplement: Supplementary file 1 [file mbo30002-0976-SD1.pdf]

*ecfG1*

*ecfG2*

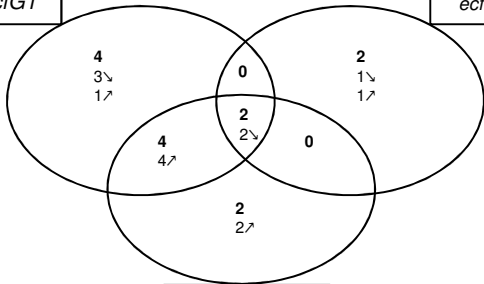

*ecfG1-ecfG2*

Supplement: Supplementary file 2 [file mbo30002-0976-SD2.pdf]
