# Supplementary material for: Canonical and non-canonical EcfG sigma factors control the general stress response in Rhizobium etli
Source: Microbiologyopen. 2013 Oct 28;2(6):976–87. doi: 10.1002/mbo3.137 (PMC3892343; doi:10.1002/mbo3.137)
Supplement: Supplementary file 3 [file mbo30002-0976-SD3.pdf]

Table S1: Bacterial strains and plasmids used in this study

Strain or plasmid	Description	Source or reference
STRAINS		
Escherichia coli		
TOP10	F^-, *mcrA* Δ(*mrr-hsdRMS-mcrBC*), Φ80*lacZ*ΔM15, Δ*lacX74*, *recA1*, *araD139*, Δ(ara-leu)7697, *galU*, *galK*, *rpsL* (Str^R), *endA1*, *nupG*	Invitrogen
JW0581	*gusA*-deficient strain used for carrying out heterologous expression experiments	(Baba et al., 2006)
Rhizobium etli		
CFN42	Nal^R wild type	(Gonzalez et al., 2006)
CMPG13304	Nal^R Sp^R *phyRtcrY*::Ω-Sp	This study
CMPG13306	Nal^R Km^R *ecfG1*::Ω-Km	This study
CMPG13308	Nal^R Km^R *ecfG2*::Ω-Km	(Vercruysse et al., 2011)
CMPG13309	Nal^R Km^R Sp^R *ecfG1*::Ω-Km *ecfG2*::Ω-Sp	(Vercruysse et al., 2011)
PLASMIDS		
pHP45ΩSp	Ap^R Sp^R	(Fellay et al., 1987)
pHP45ΩKm	Ap^R Km^R	(Fellay et al., 1987)
pCR4Blunt-TOPO	Ap^R Km^R, cloning vector	Invitrogen
pJQ200-uc1	Gm^R *sacB*, suicide vector	(Quandt and Hynes, 1993)
pRK2073	Sp^R ColE1 helper plasmid for triparental conjugation	(Figurski and Helinski, 1979)
pBAD/HisA	Ap^R, N-terminal His_6-Tag	Invitrogen
pFAJ1703	Ap^R Tc^R *gusA*, stable RK2-derived promoter-probe vector	(Dombrecht et al., 2001)
pCMPG13512	*phyR* promoter region (P*phyR*) cloned upstream of *gusA* into pFAJ1703	This study
pCMPG13513	*ecfG1* promoter region (P*ecfG1*) cloned upstream of *gusA* into pFAJ1703	This study
pCMPG13514	sRNA ReC64 promoter region (PncRNA) cloned upstream of *gusA* into pFAJ1703	This study
pCMPG13515	*ecfG2* promoter region (P*ecfG2*) cloned upstream of *gusA* into pFAJ1703	This study
pCMPG13516	*ecfG1* in pBAD/HisA	This study
pCMPG13517	*ecfG2* in pBAD/HisA	This study
pCMPG13518	pJQ200-uc1 containing *phyRtcrY*::Ω-Sp	This study
pCMPG13519	pJQ200-uc1 containing *ecfG1*::Ω-Km	This study
