# Supplementary material for: Canonical and non-canonical EcfG sigma factors control the general stress response in Rhizobium etli
Source: Microbiologyopen. 2013 Oct 28;2(6):976–87. doi: 10.1002/mbo3.137 (PMC3892343; doi:10.1002/mbo3.137)
Supplement: Supplementary file 4 [file mbo30002-0976-SD4.pdf]

Table S2: Primers used in this study

Primer name	Sequence (5’→3’)	Description
**Oligonucleotide primers**		
**SPI 0482**	CACCGCGGCCGCGGCCATCGATTCCGTGAACAGCCC	Δ*phyRtcrY* Fwd
**SPI 0483**	CACCGCGGCCGCCGGTCGCATAACGCTGCAGGC	Δ*phyRtcrY* Rev
**SPI 0484**	CACCGCGGCCGCAGAAGATTCGCAGCCAAGCCAAC	Δ*ecfG1* Fwd
**SPI 0485**	CACCGCGGCCGCGGTGGAACCAAACAGTTGCGTG	Δ*ecfG1* Rev
**SPI 1422**	GTTAACCATCAACTCGTCTTCTCACCG	*phyR-ecfG1-gusA* Fwd
**SPI 1423**	GTTAACCATCAAGGGCTCCCGTATGC	*phyR-ecfG1-gusA* Rev
**SPI 2538**	GTTTAAACCGTTCCATTGAAGTTCCC	ncRNA ReC64-*gusA* Fwd
**SPI 3050**	CACCCTCGAGGAAGAGAAACTGCAA	*ecfG1* overexpression pBAD Fwd
**SPI 3051**	ACTGAAGCTTAATCGCTTGTCAGAA	*ecfG1* overexpression pBAD Rev
**SPI 3231**	TCTAGACGATGCGATCACCATCCGGA	ncRNA ReC64-*gusA* Rev
**SPI 4317**	CACCCTCGAGGCTTCCTCGTCTTCAGCACG	*ecfG2* overexpression pBAD Fwd
**SPI 4318**	ACTGAAGCTTTCAATCGACGAGATGGTTTC	*ecfG2* overexpression pBAD Rev
**SPI 7864**	CACCGGATCCCGTTCAGAGTCTTCTCGGTTGCCA	*ecfG2-gusA* Fwd
**SPI 7865**	ACTGTCTAGAGGCCGGCGGTTGACCATAGC	*ecfG2-gusA* Rev
**RT-qPCR primers**		
**SPI 1547**	TCCACGCCGTAAACGATGA	RHE_CH00059 (16S) Fwd
**SPI 1548**	TGCGCCACCGAACAGTATAC	RHE_CH00059 (16S) Rev
**SPI 2335**	TCGAGCGCCTTCTTGAAATC	RHE_CH03273 (*ecfG1)* Fwd
**SPI 2336**	GGAATCGATGGCCATGCA	RHE_CH03273 (*ecfG1)* Rev
**SPI 2337**	AGGCCTCGCAGGAAATCTC	RHE_CH03275 (*phyR)* Fwd
**SPI 2338**	CGGTTCATCCTCGATAATCATG	RHE_CH03275 (*phyR)* Rev
**SPI 2403**	TGCGCAGCGATGATGATATC	RHE_PF00052 (*ecfG2)* Fwd
**SPI 2404**	AGTTCAGCGCCTTCAGCAA	RHE_PF00052 (*ecfG2)* Rev
**SPI 3016**	TCCGGATGGTGGGTTGGGGG	ncRNA ReC64 Fwd
**SPI 3017**	GGATGGTGATCGCATCGACCCAA	ncRNA ReC64 Rev
**SPI 3915**	CGGCAAGTTCCGCAATTT	RHE_CH04026 (*rpoH2*) Fwd
**SPI 3916**	CCGACATAGCCCTCTTGCA	RHE_CH04026 (*rpoH2*) Rev
**SPI 7819**	TCGGATTGTTTGCGACAGAA	RHE_CH01401 Fwd
**SPI 7820**	AATTTGGCGCCAGTCGATAA	RHE_CH01401 Rev
**SPI 7821**	CGGAAATGGCATCGAAGGT	RHE_CH02556 Fwd
**SPI 7822**	CGATCGTCGGTGAAAGGTTT	RHE_CH02556 Rev
**SPI 7823**	GGTTACGGCCGAGGAATTG	RHE_CH03385 Fwd
**SPI 7824**	CCGACATATAGGCCCACATGT	RHE_CH03385 Rev
**SPI 7825**	CCGAAGAGTTCAAGAAGCTCGAT	RHE_PF00004 Fwd
**SPI 7826**	CCTGCGAATCCGTCATCAG	RHE_PF00004 Rev
**SPI 7827**	ATAGGGACGAGCGGGTGAA	RHE_PF000367 Fwd
**SPI 7828**	CATGGAAAATGGCGTCGTT	RHE_PF000367 Rev
**SPI 7829**	AGGCGAACGATGAACATCCT	RHE_PF00078 Fwd
**SPI 7830**	GCTGATCCCTGCAGTCCAAA	RHE_PF00078 Rev
**SPI 7831**	GGTGTTGAGGGCCAAAGTCA	RHE_PF00264 Fwd
**SPI 7832**	CGTTCGAGTTCTCGCGTCTT	RHE_PF00264 Rev
**SPI 8284**	CGACATTCGTAACGCCAAGAT	RHE_PF00388 Fwd
**SPI 8285**	TTCCAGTTGCCGTCTTCGAT	RHE_PF00388 Rev
**SPI 8286**	TGCGCCGCTCTATCGTTT	RHE_PF00364 Fwd
**SPI 8287**	ATTGTTCGCGGCAGCAA	RHE_PF00364 Rev
**SPI 8288**	TCGCCATCTCGGTCATGAG	RHE_CH00033 Fwd
**SPI 8289**	AGGTTCATGCCGGCAATG	RHE_CH00033 Rev
**SPI 8290**	GAAGGATCACGGGCATATCAA	RHE_CH03209 Fwd
**SPI 8291**	CCAGTAACAGTGTGCGCAATG	RHE_CH03209 Rev
**SPI 8292**	TGGATTGGAGGAGCGAAGTG	RHE_PF00406 Fwd
**SPI 8293**	GTGAGGCGCCATCAATGTC	RHE_PF00406 Rev

Table S2: continued

Primer name	Sequence (5'→3')	Description
**5' RACE primers**		
**SPI 2034**	CACCAAGCTTTGGGTTGGGGGCGCGCATGTT	5' RACE gene specific inner primer ReC64
**SPI 2035**	CGTGAGTACGTCCGGATGGT	5' RACE gene specific outer primer ReC64
