# Supplementary material for: Canonical and non-canonical EcfG sigma factors control the general stress response in Rhizobium etli
Source: Microbiologyopen. 2013 Oct 28;2(6):976–87. doi: 10.1002/mbo3.137 (PMC3892343; doi:10.1002/mbo3.137)
Supplement: Supplementary file 5 [file mbo30002-0976-SD5.pdf]

Table S3: Distribution of EcfG sigma factors in completely sequenced Alpha-proteobacterial genomes.

Organism	Number of EcfG sequences
Acetobacter pasteurianus IFO 3283-01	2
Agrobacterium radiobacter K84	1
Agrobacterium sp. H13-3	2
Agrobacterium tumefaciens str. C58	3
Agrobacterium vitis S4	2
Azorhizobium caulinodans ORS 571	1
Bartonella clarridgeiae 73	1
Bartonella grahamii as4aup	1
Bartonella henselae str. Houston-1	1
Bartonella tribocorum CIP 105476	1
Beijerinckia indica subsp. indica ATCC 9039	1
Bradyrhizobium japonicum USDA 110	1
Bradyrhizobium sp. BTAi1	1
Bradyrhizobium sp. ORS278	1
Brevundimonas subvibrioides ATCC 15264	1
Brucella abortus bv. 1 str. 9-941	1
Brucella abortus S19	1
Brucella canis ATCC 23365	1
Brucella melitensis ATCC 23457	1
Brucella melitensis biovar *Abortus* 2308	1
Brucella melitensis bv. 1 str. 16M	1
Brucella microti CCM 4915	1
Brucella ovis ATCC 25840	1
Brucella pinnipedialis B2/94	1
Brucella suis 1330	1
Brucella suis ATCC 23445	1
Caulobacter crescentus CB15	2
Caulobacter crescentus NA1000	2
Caulobacter segnis ATCC 21756	1
Caulobacter sp. K31	4
Chelativorans sp. BNC1	1
Dinoroseobacter shibae DFL 12	1
Erythrobacter litoralis HTCC2594	1
Gluconacetobacter diazotrophicus PAl 5	4
Gluconacetobacter xylinus NBRC 3288	2
Gluconobacter oxydans 621H	2
Granulibacter bethesdensis CGDNIH1	1
Hirschia baltica ATCC 49814	1
Hyphomicrobium denitrificans ATCC 51888	2
Hyphomicrobium sp. MC1	3
Hyphomonas neptunium ATCC 15444	2

continued

Jannaschia sp. CCS1	1
Ketogulonicigenium vulgare Y25	3
Mesorhizobium ciceri biovar *biserrulae* WSM1271	2
Mesorhizobium loti MAFF303099	1
Mesorhizobium opportunistum WSM2075	2
Methylobacterium chloromethanicum CM4	5
Methylobacterium extorquens AM1	6
Methylobacterium extorquens DM4	5
Methylobacterium extorquens PA1	5
Methylobacterium nodulans ORS 2060	8
Methylobacterium populi BJ001	5
Methylobacterium radiotolerans JCM 2831	7
Methylobacterium sp. 4-46	10
Methylocella silvestris BL2	1
Nitrobacter hamburgensis X14	1
Nitrobacter winogradskyi Nb-255	1
Novosphingobium aromaticivorans DSM 12444	1
Novosphingobium sp. PP1Y	2
Ochrobactrum anthropi ATCC 49188	3
Oligotropha carboxidovorans OM5	2
Paracoccus denitrificans PD1222	2
Parvibaculum lavamentivorans DS-1	1
Parvularcula bermudensis HTCC2503	1
Pelagibacterium halotolerans B2	1
Phenylobacterium zucineum HLK1	2
Polymorphum gilvum SL003B-26A1	1
Rhizobium etli CFN 42	2
Rhizobium etli CIAT 652	2
Rhizobium leguminosarum bv. *trifolii* WSM1325	4
Rhizobium leguminosarum bv. *trifolii* WSM2304	2
Rhizobium leguminosarum bv. *viciae* 3841	1
Rhodobacter capsulatus SB 1003	1
Rhodobacter sphaeroides 2.4.1	2
Rhodobacter sphaeroides ATCC 17025	2
Rhodobacter sphaeroides ATCC 17029	2
Rhodobacter sphaeroides KD131	2
Rhodomicrobium vannielii ATCC 17100	1
Rhodopseudomonas palustris BisA53	1
Rhodopseudomonas palustris BisB18	1
Rhodopseudomonas palustris BisB5	1
Rhodopseudomonas palustris CGA009	1
Rhodopseudomonas palustris DX-1	1
Rhodopseudomonas palustris HaA2	1
Rhodopseudomonas palustris TIE-1	1
Rhodospirillum rubrum ATCC 11170	1

continued

Roseobacter denitrificans OCh 114	2
Roseobacter litoralis Och 149	2
Ruegeria sp. TM1040	1
Sinorhizobium fredii NGR234	2
Sinorhizobium medicae WSM419	2
Sinorhizobium meliloti 1021	2
Sinorhizobium meliloti AK83	2
Sphingobium chlorophenolicum L-1	1
Sphingobium japonicum UT26S	1
Sphingobium sp. SYK-6	1
Sphingomonas wittichii RW1	1
Sphingopyxis alaskensis RB2256	2
Starkeya novella DSM 506	1
Xanthobacter autotrophicus Py2	1
Zymomonas mobilis subsp. *mobilis* NCIMB 11163	1
Zymomonas mobilis subsp. *mobilis* ZM4	1
Zymomonas mobilis subsp. *pomaceae* ATCC 29192	1

Data retrieved from MiST database (http://mistdb.com; Ulrich and Zhulin, 2010) on November 29th 2012.
