# Supplementary material for: Canonical and non-canonical EcfG sigma factors control the general stress response in Rhizobium etli
Source: Microbiologyopen. 2013 Oct 28;2(6):976–87. doi: 10.1002/mbo3.137 (PMC3892343; doi:10.1002/mbo3.137)
Supplement: Supplementary file 6 [file mbo30002-0976-SD6.pdf]

Table S4: The differentially expressed genes and ncRNAs in Δ*ecfG1*, Δ*ecfG2* and Δ*ecfG1*Δ*ecfG2* compared to the wild type. Genes preceded by the EcfG1 consensus motif are shown in bold face and underlined. Genes and ncRNAs previously identified as (p)ppGpp-dependent for expression, are marked with an asterisk.

Locus tag	Operon member[a]	Gene symbol	Expression ratio (Log_2) mutant vs Wild type			Product name
			Δ*ecfG1*	Δ*ecfG2*	Δ*ecfG1*Δ*ecfG2*	
RHE_CH00014		-	-0,82	-0,31	-1,64	hypothetical protein
RHE_CH00025		*trxA1*	-1,20	-0,40	0,10	thioredoxin protein
RHE_CH00033		-	1,26	0,87	0,59	protein-N(pi)-phosphohistidine-sugar phosphotransferase protein (component of thephosphoenolpyruvate-dependent sugar phosphotransferasesystem)
RHE_CH00051		-	-1,08	-0,21	-1,53	cytochrome p450 protein
RHE_CH00111		*pnp*	-1,02	-0,71	-1,06	polynucleotide phosphorylase/polyadenylase
**<u>RHE_CH00180</u>**		-	-3,15	-0,36	-3,69	hypothetical protein
RHE_CH00247		-	-2,55	0,28	-2,78	hypothetical protein
RHE_CH00252		-	-0,63	-0,81	-1,76	haloacid dehalogenase-like hydrolase
RHE_CH00256		-	-0,52	-1,01	-1,24	hypothetical protein
RHE_CH00257		-	-0,26	-1,07	-0,73	hypothetical protein
**<u>RHE_CH00268</u>**		-	-3,73	0,08	-4,13	hypothetical protein
RHE_CH00303		-	-0,62	-0,53	-1,10	hypothetical protein
RHE_CH00304		-	0,84	0,76	1,05	hypothetical protein
RHE_CH00306		-	1,10	0,20	1,10	rare lipoprotein A protein
RHE_CH00398		-	-1,27	-1,51	-0,96	hypothetical protein
RHE_CH00407		*ptsNch*	-1,18	-0,95	-0,54	protein-N(pi)-phosphohistidine-sugar phosphotransferase protein (enzyme II of thephosphotransferase system)
**<u>RHE_CH00479</u>**		-	-2,95	-0,09	-3,69	hypothetical protein
RHE_CH00493		*fucUch2*	-0,99	-0,93	-1,26	fucose dissimilation pathway protein
	RHE_CH00494	-	-0,85	-0,91	-1,09	L-fucose dehydrogenase protein
RHE_CH00495		-	-0,90	-0,84	-1,19	altronate hydrolase protein
**<u>RHE_CH00521</u>**		*apaG*	-1,15	0,54	-0,88	ApaG
RHE_CH00543		-	0,97	0,21	1,13	hypothetical protein
RHE_CH00545		-	0,90	-0,10	1,01	hypothetical protein
RHE_CH00630		-	-1,07	-0,07	-1,70	putative two-component sensor histidine kinase protein
RHE_CH00723		-	0,05	1,03	-0,32	metallopeptidase protein
RHE_CH00777		-	-0,94	-0,61	-1,61	insertion sequence transposase protein
RHE_CH00778		-	-1,01	-0,61	-1,69	insertion sequence transposase protein

RHE_CH00801		-	1,00	0,86	0,82	hypothetical protein
RHE_CH00813		-	1,21	0,68	1,50	hypothetical protein
**RHE_CH00851**[*]		-	-4,39	-0,04	-4,70	hypothetical protein
RHE_CH00927		-	-1,58	-0,30	-1,67	putative acetyltransferase protein
RHE_CH00954		*ctaB*	-0,34	-1,07	-0,31	protoheme IX farnesyltransferase
RHE_CH00985		-	-2,08	-0,27	-2,47	cytochrome-c oxidase protein
RHE_CH01067		-	0,93	1,00	0,77	hypothetical protein
RHE_CH01129		-	-0,58	-1,39	-0,64	hypothetical protein
RHE_CH01139[*]		-	-2,15	-0,34	-2,81	hypothetical protein
RHE_CH01141		-	-0,83	-0,99	-1,28	quinone oxidoreductase protein
RHE_CH01165		-	-0,48	-0,75	-1,22	putative aminopeptidase protein
RHE_CH01199		-	-0,54	0,56	-1,19	hypothetical protein
RHE_CH01222		*gcd*	-2,01	-0,93	-2,48	glucose dehydrogenase (pyrroloquinoline-quinone) protein
RHE_CH01228		-	-0,68	-1,07	-1,62	putative polysaccharide deacetylase protein
**RHE_CH01231**[*]		-	-2,30	0,41	-2,83	hypothetical protein
RHE_CH01262		-	-0,97	0,07	-1,08	hypothetical protein
RHE_CH01277		*gstR*	-0,97	-1,09	-1,93	LysR family transcriptional regulator
RHE_CH01278		*gstA*	-0,90	-0,99	-1,57	glutathione S-transferase protein
RHE_CH01310		-	-1,22	-1,33	-0,76	putative permease protein
RHE_CH01312		*mgtE*	-1,09	-1,65	-0,71	magnesium (mg2+) transporter protein
RHE_CH01333[*]		-	-1,09	-0,89	-0,58	hypothetical protein
RHE_CH01440		*rpsF*	1,15	0,76	0,96	30S ribosomal protein S6
RHE_CH01527		*hppA*	0,46	-0,16	1,04	membrane-bound proton-translocating pyrophosphatase
RHE_CH01570		*rpsI*	0,72	0,53	1,18	30S ribosomal protein S9
	RHE_CH01571	*rplM*	0,91	0,35	1,35	50S ribosomal protein L13
RHE_CH01670		*rpsL*	1,33	0,47	1,14	30S ribosomal protein S12
RHE_CH01674		*rpsJ*	1,02	0,34	1,07	30S ribosomal protein S10
	RHE_CH01675	*rplC*	1,13	0,64	1,09	50S ribosomal protein L3
RHE_CH01679		*rpsS*	1,10	0,74	1,03	30S ribosomal protein S19
RHE_CH01681[*]		*rpsC*	1,00	0,08	1,29	30S ribosomal protein S3
RHE_CH01682[*]		*rplP*	1,12	0,33	0,98	50S ribosomal protein L16
RHE_CH01684		*rpsQ*	1,13	0,37	0,85	30S ribosomal protein S17
RHE_CH01685[*]		*rplN*	1,26	0,06	1,17	50S ribosomal protein L14
RHE_CH01688[*]		*rpsN*	0,75	-0,61	1,11	30S ribosomal protein S14

RHE_CH01691*		*rplR*	0,82	-0,40	1,05	50S ribosomal protein L18
	RHE_CH01692*	*rpsE*	0,90	0,70	1,06	30S ribosomal protein S5
RHE_CH01697		*rpsM*	1,30	0,02	1,06	30S ribosomal protein S13
RHE_CH01699*		*rpoA*	1,24	0,53	0,93	DNA-directed RNA polymerase subunit alpha
RHE_CH01715*		-	-2,56	-0,46	-2,76	hypothetical protein
**RHE_CH01778***		-	-1,51	-0,30	-1,55	hypothetical protein
RHE_CH02063		*dppDch2*	-0,78	-0,12	-1,10	dipeptide ABC transporter, ATP-binding protein
RHE_CH02064		*dppFch2*	-0,71	-0,27	-1,00	dipeptide ABC transporter, ATP-binding protein
RHE_CH02085		-	1,06	0,79	0,98	sugar ABC transporter, substrate-binding protein
RHE_CH02153*		-	-1,81	-0,12	-1,97	hypothetical protein
**RHE_CH02172***		-	-2,93	-0,91	-3,31	hypothetical protein
RHE_CH02591*		-	-0,50	0,35	-1,22	hypothetical protein
RHE_CH02605		*plyA1*	-1,13	-0,65	-1,21	polysaccharidase protein
RHE_CH02606*		*plyA2*	1,57	1,36	1,65	polysaccharidase protein
**RHE_CH02625***		-	-1,05	-0,37	-1,05	hypothetical protein
**RHE_CH02629***		-	-1,77	-0,23	-2,07	hypothetical protein
RHE_CH02687		-	-1,11	-0,78	-1,02	hypothetical protein
**RHE_CH02761***		-	-0,97	-0,11	-1,18	hypothetical protein
RHE_CH02763		-	-0,99	-1,31	-1,18	aspartate racemase protein
RHE_CH02796		*hflC*	0,35	-0,50	1,37	hydrolase serine protease transmembrane subunit C protein
RHE_CH02992*		-	-1,48	-0,49	-1,07	hypothetical protein
RHE_CH03000		-	0,56	0,29	1,10	hypothetical protein
RHE_CH03047*		-	-1,43	-0,89	-0,98	hypothetical protein
**RHE_CH03048***		-	-1,46	-1,21	-1,21	hypothetical protein
RHE_CH03056		-	-1,03	-0,68	-1,72	insertion sequence transposase protein
RHE_CH03147		-	-0,07	1,12	-0,11	hypothetical protein
RHE_CH03185*		-	0,39	1,01	-0,20	hypothetical protein
RHE_CH03187*		-	-1,19	-0,06	-1,81	hypothetical protein
RHE_CH03209		-	1,90	0,93	1,28	putative methyltransferase protein
RHE_CH03263		-	1,22	1,99	1,15	hypothetical protein
RHE_CH03268		*hmuS*	1,15	1,63	1,05	hemin transport system, degrading protein
**RHE_CH03272***		-	-3,92	-0,79	-4,61	hypothetical protein
**RHE_CH03274**		-	-2,65	-0,06	-2,94	hypothetical protein
	RHE_CH03273*	*rpoE4/ecfG1*	-2,81	-0,01	-3,25	RNA polymerase sigma factor

**RHE_CH03275***	*tcrX/phyR*	-5,08	-0,07	-4,96	two-component response regulator
RHE_CH03278*	-	-0,67	-0,20	-1,01	dihydropyrimidine dehydrogenase
RHE_CH03357	-	-0,99	-1,28	-1,79	HlyD family protein secretion protein
RHE_CH03360	*mexE2*	0,92	0,05	1,06	HlyD family multidrug-efflux system secretion protein
RHE_CH03393*	-	0,90	0,50	1,17	acetyltransferase protein
RHE_CH03430	-	0,56	-0,41	1,09	hypothetical protein
RHE_CH03442	-	-1,87	-0,28	-2,14	putative phosphoketolase
**RHE_CH03453***	-	-3,94	-0,45	-4,65	hypothetical protein
**RHE_CH03474***	-	-3,33	0,47	-3,89	hypothetical protein
RHE_CH03495*	*tkt*	0,98	0,04	1,23	transketolase
RHE_CH03508	-	-2,69	-0,77	-2,73	hypothetical protein
RHE_CH03555	-	-1,40	-0,13	-1,22	5`-nucleotidase protein
RHE_CH03582	-	-1,95	-1,05	-2,15	hypothetical protein
RHE_CH03584*	-	-3,19	-0,94	-2,82	hypothetical protein
RHE_CH03583*	-	-3,01	-0,24	-2,92	hypothetical protein
RHE_CH03736	*nuoA2*	-0,67	-0,38	-1,09	NADH-ubiquinone oxidoreductase chain A protein
RHE_CH03774	-	-1,24	-0,41	-1,55	oxidoreductase (aldo/keto reductase) protein
RHE_CH03885	-	-0,67	-0,10	-1,38	hypothetical protein
RHE_CH03886	-	-0,88	-0,22	-1,28	hypothetical protein
RHE_CH03925	-	-1,09	-0,24	-0,73	oxidoreductase protein
RHE_CH04006*	-	-0,94	-0,48	-1,11	amino acid ABC transporter substrate-binding protein
**RHE_CH04021***	-	-1,71	-0,68	-1,29	hypothetical protein
**RHE_CH04026**	*rpoH2*	-3,76	-0,34	-4,36	RNA polymerase factor sigma-32
RHE_PA00140	-	-1,21	-0,73	-1,16	hypothetical protein
RHE_PB00114*	-	-1,26	-0,62	-1,11	putative sugar ABC transporter, permease protein
RHE_PB00154	-	-0,48	-1,11	-0,14	oxidoreductase protein
RHE_PC00065	-	-0,51	-0,60	-1,00	hypothetical protein
RHE_PC00075	-	-1,01	-0,94	-1,19	stomatin-like protein
RHE_PC00093*	-	-0,69	1,01	-1,11	hypothetical protein
RHE_PC00114	-	-0,91	-1,05	-0,18	hypothetical protein
RHE_PC00228*	*otsB*	-3,19	-0,49	-3,62	trehalose-phosphatase protein
RHE_PD00101	-	-0,93	-0,59	-1,55	insertion sequence transposase protein
RHE_PD00114*	-	0,59	1,02	-0,08	4-hydroxyphenylacetate-3-monooxygenase protein
RHE_PD00127	-	-0,76	0,18	-1,23	phosphoglycerate mutase protein

RHE_PD00192		-	-1,15	-1,07	-0,92	carbonic anhydrase protein
RHE_PD00347		-	-0,95	-0,62	-1,54	insertion sequence transposase protein
RHE_PE00027		*cyoD*	-1,12	-1,11	-1,32	cytochrome O ubiquinol oxidase, subunit IV protein
RHE_PE00031		-	-0,66	-1,22	-0,22	putative ABC transporter, permease protein
RHE_PE00171		-	-1,02	0,12	-0,68	hypothetical protein
RHE_PE00198[*]		-	0,32	-0,43	-1,17	putative succinoglycan biosynthesis transport protein
RHE_PE00221		-	-1,28	-1,14	-1,52	hypothetical protein
	RHE_PE00220	-	-1,10	-0,46	-1,64	hypothetical protein
RHE_PE00374[*]		-	-0,67	-1,26	-0,54	hypothetical protein
RHE_PE00376		-	-0,90	-0,99	-1,08	hypothetical protein
RHE_PF00004		*katG*	-0,60	-1,09	-0,42	catalase protein
**RHE_PF00044**[*]		-	-1,73	0,28	-1,62	hypothetical protein
**RHE_PF00051**[*]		-	-0,98	0,33	-1,09	hypothetical protein
RHE_PF00068[*]		-	0,32	1,18	0,36	C4-dicarboxylate ABC transporter, substrate-binding protein
**RHE_PF00085**[*]		-	-2,28	-0,17	-2,85	hypothetical protein
RHE_PF00086		-	-0,98	-0,08	-1,23	putative NTP pyrophosphohydrolase protein
RHE_PF00260		-	-0,64	0,34	-1,51	hypothetical protein
**RHE_PF00261**		-	-0,63	0,21	-1,52	hypothetical protein
RHE_PF00265		-	-3,03	-0,60	-3,46	two-component sensor histidine kinase/response regulator hybrid protein
RHE_PF00267[*]		-	-1,47	0,24	-1,86	hypothetical protein
RHE_PF00364		*gabDf2*	-0,83	2,07	0,22	succinate-semialdehyde dehydrogenase protein
RHE_PF00367		-	-0,30	1,86	0,09	GntR family transcriptional regulator
RHE_PF00368		-	0,34	1,01	0,11	hypothetical protein
RHE_PF00388		-	-0,11	1,24	0,68	putative dyoxygenase (alpha subunit) oxidoreductase protein
RHE_PF00406		-	0,08	1,21	-0,12	putative transcriptional regulator protein
RHE_PF00534		-	-0,40	-1,39	-0,48	aldehyde dehydrogenase
ReC06[*]		-	-2,42	-1,74	-1,15	ncRNA
ReC10		-	1,25	0,23	1,44	ncRNA
ReC14[*]		-	-1,01	-0,77	0,47	ncRNA
ReC29[*]		-	0,61	-0,54	1,09	ncRNA
ReC31		-	1,55	0,53	1,50	ncRNA
ReC33		-	1,38	0,40	0,51	ncRNA
ReC34		-	1,17	0,45	1,03	ncRNA

ReC35	-	0,46	1,09	0,86	ncRNA
ReC55*	-	-2,07	-0,41	-0,38	ncRNA
ReC64*	-	-4,77	-1,01	-5,01	ncRNA
ReC68	-	1,07	0,46	1,19	ncRNA
ReC69	-	0,44	-0,55	1,03	ncRNA
ReC70*	-	-1,94	-0,75	-0,39	ncRNA
ReC76*	-	0,81	-1,01	0,49	ncRNA

[a] based on the signal from the different probes used in transcriptome analyses
